# The Cleft Care UK study. Part 4: perceptual speech outcomes

**DOI:** 10.1111/ocr.12112

**Published:** 2015-11-16

**Authors:** D Sell, S Mildinhall, L Albery, A K Wills, J R Sandy, A R Ness

**Affiliations:** Speech and Language Therapy Department and Centre for Outcomes and Experience Research in Children’s Health, Illness and Disability (ORCHID), Great Ormond Street Hospital NHS Foundation TrustLondon, UK; Previously South Thames Cleft Service, Guys and St Thomas’ NHS Foundation Trust HospitalLondon, UK; University Hospitals Bristol NHS Trust, Cleft Lip and Palate TeamBristol, UK; School of Oral and Dental Sciences, University of BristolBristol, UK; National Institute for Health Research (NIHR) Biomedical Research Unit in Nutrition, Diet and Lifestyle at the University Hospitals Bristol NHS Foundation Trust and the University of BristolBristol, UK

**Keywords:** cleft lip, cleft palate, speech, treatment outcome

## Abstract

**Objectives:**

To describe the perceptual speech outcomes from the Cleft Care UK (CCUK) study and compare them to the 1998 Clinical Standards Advisory Group (CSAG) audit.

**Setting and sample population:**

A cross-sectional study of 248 children born with complete unilateral cleft lip and palate, between 1 April 2005 and 31 March 2007 who underwent speech assessment.

**Materials and methods:**

Centre-based specialist speech and language therapists (SLT) took speech audio–video recordings according to nationally agreed guidelines. Two independent listeners undertook the perceptual analysis using the CAPS-A Audit tool. Intra- and inter-rater reliability were tested.

**Results:**

For each speech parameter of intelligibility/distinctiveness, hypernasality, palatal/palatalization, backed to velar/uvular, glottal, weak and nasalized consonants, and nasal realizations, there was strong evidence that speech outcomes were better in the CCUK children compared to CSAG children. The parameters which did not show improvement were nasal emission, nasal turbulence, hyponasality and lateral/lateralization.

**Conclusion:**

These results suggest that centralization of cleft care into high volume centres has resulted in improvements in UK speech outcomes in five-year-olds with unilateral cleft lip and palate. This may be associated with the development of a specialized workforce. Nevertheless, there still remains a group of children with significant difficulties at school entry.

## Introduction

During the 1980s, the Eurocleft study showed that many aspects of care and some outcomes of treatment in two UK centres fell below those of European Centres such as Oslo in Norway 1. The Clinical Standards Advisory Group (CSAG) study determined multidisciplinary outcomes of children born with unilateral complete cleft lip and palate (UCLP) at the ages of five and twelve years 2–5. Some outcomes were poor, and this included speech 4. They reported that the speech in 19% of five-year-olds and 4% of twelve-year-olds was judged to be impossible to understand or only just intelligible to strangers. Thirty-four per cent of five-year-olds and 17% of twelve-year-olds had at least one serious error of consonant production. Eighteen per cent of five-year-olds and twelve-year-olds had consistent hypernasality of mild, moderate or severe degree.

The centralization of services after the publication of the CSAG report means that most centres treat more than 60 new cleft babies each year [range, 45 (Northern Ireland) to 151 (North Thames)] 6. The increased numbers of children treated in each centre have also supported the development of a specialized workforce in all disciplines. More meaningful audit of outcomes is also possible with the larger numbers treated in each centre. In response to these audit requirements, the Cleft Audit Protocol for Speech–Augmented (CAPS-A) was developed and validated as a tool for speech audit studies 7. More recently, NHS England (2013) has produced a National Service Specification for Cleft Lip and/or Palate services which is a framework for uniformity of care provision 8.

By the age of five years, the expectation in the non-cleft population is that the acquisition of speech sounds should be more or less complete 9. This is also the time at which children in the UK go to primary school with a target that they should have ‘normal’ speech, which does not draw comment from their peers or teachers. However, children with cleft palate ± lip are at high risk of speech difficulties 10–12. These can be broadly described in two problem areas. The first are structurally related difficulties which include hypernasality, nasal airflow (abnormal nasal emission or nasal turbulence on consonant sounds), weak nasalized consonants and the excessive use of nasal consonants. The latter are known as passive characteristics 7,13. These speech characteristics are usually the result of velopharyngeal insufficiency (VPI) and/or a fistula after primary palate repair. A percentage of children who have their cleft palate repaired will have velopharyngeal insufficiency, but this is not predictable to the individual 14–16.

The second possible speech difficulty is the incorrect production of speech sounds (consonants) referred to as cleft speech characteristics (CSCs). These can be divided into anterior oral CSCs, for example palatal or lateral errors, posterior oral CSCs where sounds are produced further back in the oral cavity, for example /t/ is produced as /k/, non-oral CSCs where consonant sounds are produced even further back in the larynx, pharynx or velopharynx. Many of these, such as glottal and pharyngeal CSCs, may be the result of early mislearning and are often associated with velopharyngeal insufficiency. Such errors persist even after successful secondary speech surgery and speech therapy intervention is required.

These speech disorders frequently reduce intelligibility and acceptability 17. When such speech disorders persist beyond 5 years of age, there can be far-reaching consequences for communication, literacy and psychosocial well-being 18–21. Secondary speech surgery and/or fistula closure is usually needed to correct structurally related speech difficulties. Speech therapy is needed to address some of the anterior, posterior and non-oral cleft speech characteristics. Children with cleft palate are also at risk of speech disorders for other reasons, such as intermittent conductive hearing loss, most typically caused by glue ear 22,23. In addition, they are vulnerable, like any other child to other factors such as the lack of a stimulating environment, family history of speech difficulties and expressive language delay, which can also impact speech performance at 5 years.

The aim of this study was to determine the perceptual speech outcomes of children included in the CCUK study and to compare these with speech outcomes reported in the CSAG study 5.

## Materials and methods

### Study design and population

Details of the recruitment and selection of children into this study can be found elsewhere 24. Two hundred and fifty-four children underwent digital speech video–audio recording; of these, six could not be analysed. One participant refused to speak, and five recordings were technically too poor to analyse. The final analysis thus comprised 248 children with speech recordings (93% of all participants). The CSAG study was similar in design and target population and was conducted as a cross-sectional audit in 1996–1997. We have compared the CCUK data with the original CSAG survey published in 1998 which shares a similar design and target population – it was cross-sectional and also attempted to locate and study all five-year-old children with non-syndromic UCLP, but born between 1 April 1989 and 31 March 1991. Further details about CSAG are available elsewhere 2. Data on speech parameters 5 were used to compare outcomes of CSAG children with the CCUK children (pre- versus post-centralization of cleft services).

### Procedures and equipment

Each child was seen individually with a parent or carer by a centre-based SLT, and speech audio–video recordings were undertaken according to nationally agreed guidelines 7,25. These are detailed in the methods section of this supplement 26. Variation in the recording equipment is shown in Appendix S1. Each team made individual arrangements for the copying of the DV recordings to a DVD. The original recordings remained at each centre, and the copy was passed to the CCUK research team at Bristol. Each sample was assigned a number for subsequent analysis by independent listeners. Names were edited from recordings to provide anonymity to the child. All the samples were placed in a randomized order onto an encrypted external hard drive (iStorage Disk Ashur).

### Outcomes and analysis

The CAPS-A tool has six non-articulation outcome parameters of intelligibility/distinctiveness, hypernasality, hyponasality, audible nasal emission and nasal turbulence. Intelligibility/distinctiveness and hypernasality are rated on ordinal rating scales of five scalar points, whereas all the other scales consist of three scalar points. The articulation parameters are based on narrow phonetic transcription which is then coded into CSCs. Scoring of each CSC is captured according to the number of target consonants affected by the characteristic. A score of 0 represents an absence of the CSC, 1 represents where there are 1 or 2 target consonants produced as the error type, and 2 represents where there are 3 or more target consonants affected. The individual cleft speech characteristics are summarized within the four categories of anterior oral CSCs, posterior oral CSCs, non-oral CSCs and passive CSCs each on a 3-point rating scale. The tool also captures non-cleft speech immaturities on a binary scale.

Two independent listeners were employed to undertake the perceptual analysis. The listeners undertook the analysis using Sennheiser DT100 headphones. Both listeners had undergone CAPS-A training 25 and had previously reported intra- and inter-rater reliability as good/excellent. Before analysis, the two SLTs underwent a familiarization and revision session with one of the authors to ensure consistency in listening. The first phase of the analysis, the inter-rater reliability study, was based on 80 samples. Thirty per cent (N = 24) were randomly redistributed within the overall sample to provide intrarater reliability. After the completion of the reliability study, the remaining samples were analysed.

### Statistical analysis

To describe and quantify intra- (N = 24) and inter-rater reliability (N = 80) of the CAPS-A tool, we calculated the percentage of scores that were in complete agreement and estimated weighted (linear) kappa coefficients for all speech parameters (described above) except the binary variable non-cleft speech immaturities which naturally cannot be weighted. Intra-observer reliability was assessed for each of the two observers using data from the 24 repeated measurements, and interobserver reliability was assessed using readings taken by both observers.

In accordance with recommendations from the Scandcleft Group 26, we used readings from the second observer to describe and compare outcomes in the CCUK because they showed better intrarater reliability. Relative frequencies for each outcome were calculated and where data allowed, a comparison with the CSAG study was performed using previously reported data for the CSAG study 4. Relative risk ratios (RR) were estimated using CSAG as the reference category, and 95% confidence intervals and *p*-values were estimated using the normal approximation.

## Results

The median age of the CCUK children with speech assessment (N = 248) was 5.5 years (IQR: 5.4–5.7). The majority were boys (67.3%).

### Reliability

Table1 shows the intra- and inter-rater reliability results. For intrarater reliability, percentage agreement was over 82% for both listeners on all parameters, with a mean exact agreement of 91% for Listener 1 and 96% for Listener 2. Listener 1 had substantial to almost perfect agreement (range of *κ*: 0.65–0.89) for all parameters except for hyponasality and non-cleft speech immaturities which had moderate agreement. Listener 2 had a very high percentage of intrarater agreement in scores (range of 88–100%), and substantial to almost perfect agreement as indicated by the kappa coefficients (range: 0.65–1.0) for all parameters.

**Table 1:** Intra- (N = 24) and Inter-rater (N = 80) Reliability as expressed using % agreement and weighted kappa (*κ*)

Parameter	Intrarater: Listener 1		Intrarater: Listener 2		Inter-rater	
	% agreement	*κ*	% agreement	*κ*	% agreement	*κ*
Intelligibility 5-point scale	90	0.65	88	0.65	78	0.52
Hypernasality 5-point scale	93	0.69	100	1.00	92	0.60
Hyponasality 3-point scale	88	0.46	100	1.00	95	0.67
Nasal Emission 3-point scale	96	0.87	96	0.85	84	0.46
Nasal Turbulence 3-point scale	90	0.73	94	0.83	90	0.69
Anterior 3-point scale	90	0.66	98	0.89	81	0.51
Posterior 3-point scale	98	0.89	98	0.84	90	0.54
Non-oral 3-point scale	96	0.71	99	0.77	88	0.36
Passive 3-point scale		†		†	95	0.60
Non-cleft speech immaturities Binary scale	82	0.58	92	0.81	62	0.30

† It was not possible to calculate a kappa for the intrarater agreement because of the structure of the data – 22 of 24 were rated A on both occasions and the other 2 pairs of measurements were discordant.

The percentage agreement for inter-rater reliability was more than 78%, except for non-cleft speech immaturities with a mean exact agreement of 62%. The parameters of hyponasality and nasal turbulence had substantial agreement. Hypernasality and passive all fall on the cusp of the substantial and moderate categories of agreement. Moderate agreement is found on the parameters of hyponasality, anterior and posterior categories and fair agreement on the non-oral category and non-cleft speech immaturities.

### Speech outcomes

Table2 describes the prevalence of outcomes for each of the speech parameters. There was strong evidence for a reduction in the prevalence of hypernasality comparing CCUK against CSAG children (RR = 0.58; 95% CI: 0.36, 0.90). There was no significant difference in nasal emission outcomes and evidence that turbulence and hyponasality were more prevalent among the CCUK children. Table2 also describes the CSCs in the CSAG and CCUK children. There was evidence for a reduction in the prevalence of the following: palatal/palatalization; backed to velar/uvular; glottal; weak and nasalized consonants; and nasal realizations *χ*^2^(1) = 8.6784 *p* < 0.003. The only CSC where there was evidence for a higher prevalence in CCUK children was lateral/lateralization. Backed to velar/uvular was a common error category for both data sets, with 14.9% of children showing this error type in the CCUK data, half of whom had more than three targets affected. Lateral/lateralization was also prevalent in the CCUK data set and 17.3% of children had this error type; over half of whom of whom have more than three targets affected. In the CSAG data set, palatal/palatalization was the commonest cleft speech characteristic with a striking reduction of this characteristic in the CCUK data set. Weak and nasalized consonants were also prevalent in the CSAG data set, in contrast to the CCUK study, indicating structural problems. Table2 also shows these data as summary categories of the individual cleft speech characteristics. There was strong evidence for a reduction in each category in the CCUK children compared to CSAG, although the anterior category was not directly comparable due to the omission of the dentalization/interdentalization CSC in CCUK. The more severe categories of non-oral (pharyngeal, glottal, active nasal fricatives) and passive cleft speech characteristics (weak and nasalized, nasal realizations and gliding) had a very low prevalence in the CCUK data set: only 10% had non-oral errors and 7% had passive errors.

**Table 2:** Summary of the prevalence of outcomes of the speech parameters in CCUK and a comparison with the CSAG children (Risk Ratio)

Speech parameter	CCUK		CSAG		Risk Ratio (95% CI)	*p*-value
	N	(%)	N	(%)		
Presence of:						
Hypernasality	25/245	10.2%	42/238	17.6%	0.58 (0.36–0.92)	0.018
Nasal emission	36/247	14.6%	46/238	19.3%	0.75 (0.51–1.12)	0.16
Turbulence	75/247	30.4%	30/238	12.6%	1.85 (1.25–2.73)	0.001
Hyponasality	30/248	12.1%	8/238	3.4%	3.6 (1.68–7.7)	<0.001
Presence of:						
Palatal	20/248	8.1%	90/238	37.8%	0.21 (0.14–0.33)	<0.001
Lateral	43/248	17.3%	24/238	10.1%	1.72 (1.08–2.74)	0.02
Backed to velar/uvular	37/248	14.9%	71/238	29.8%	0.50 (0.35–0.71)	<0.001
Glottal	12/248	4.8%	36/238	15.1%	0.32 (0.17–0.60)	<0.001
ANF	14/248	5.6%	21/217	8.8%	0.64 (0.33–1.22)	0.18
Weak or nasalized	11/248	4.4%	50/238	21.0%	0.21 (0.11–0.40)	<0.001
Nasal realizations	10/248	4.0%	26/238	10.9%	0.37 (0.18–0.75)	<0.001
Speech summary patterns:						
Anterior	58/248	23.3	81/236	34.3	0.68 (0.51–0.91)	0.008
Posterior	36/247	14.6	67/236	28.3	0.51 (0.36–0.74)	<0.001
Non-oral	25/248	10.1	41/236	17.4	0.58 (0.36–0.92)	0.02
Passive	18/247	7.2	48/236	20.3	0.36 (0.21–0.60)	<0.001

### Intelligibility/distinctiveness

**Figure 1:**
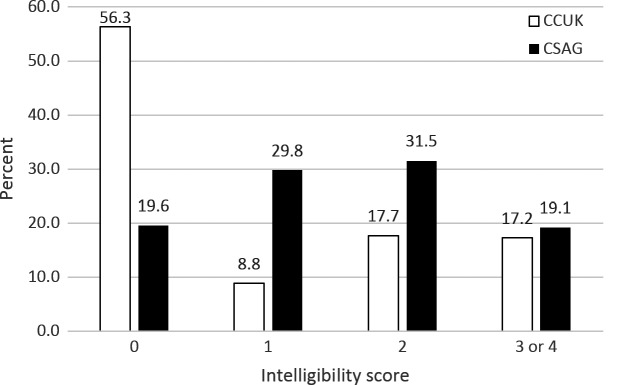
Distribution of intelligibility scores among five-year-old children in the CCUK (N = 238) and CSAG study (N = 235).

Figure1 describes the outcome of intelligibility/distinctiveness. There was a striking difference in the percentage of children rated as ‘normal’, with 19.6% falling into this category for the CSAG study compared with 56.3% for CCUK (*p* < 0.001). It is also of note that just under 20% of both data sets have speech falling in the two most severe categories defined as ‘only just intelligible to strangers’ or ‘impossible to understand’.

### Age associations (secondary analysis)

The CSAG children were slightly older compared to CCUK children. It is known that speech improves with advancing age 27,28. We therefore fitted logistic and ordinal regression models to estimate the association of age with each speech parameter in the CCUK children. Within the narrow range of ages in the CCUK study, there was no evidence for an association with age in for any of the speech parameters in this study.

## Discussion

The aim of this investigation was to determine the impact of the centralization of cleft palate services on speech outcomes, through comparison of the original CSAG study with CCUK. For the majority of speech parameters, there was evidence to suggest that speech outcomes have improved following the reorganization. There were some differences between the two studies. For example, the cleft speech characteristic of dentalization/interdentalization was omitted from the CCUK data set, in contrast to CSAG, as there has been controversy about this classification. This is because it can be associated with a Class III malocclusion in children with cleft palate but is also common in non-cleft five-year-olds where it is considered a developmental immaturity. Special mention should also be made of the intelligibility/distinctiveness scale which was in the original CAPS. This had good reliability in the CSAG study and has been shown in other studies to have good inter-rater reliability 25,29. This global measure is designed to assist the interpretation of the results of the individual parameters but should never be used as a ‘stand alone’ speech result 30. However, more recently, intelligibility/understandability and acceptability have been considered as two separate entities 31. In the CAPS/CAPS-A scales, these two parameters are collapsed into one scale with doubts on its validity 32. Intelligibility is a complex speech parameter with controversies about definition, its measurement, the stimulate used for assessment and who is the most appropriate judge, be it layman or professional 31,33. The scale has now been removed from routine audit reporting in the UK but has been included in this study to enable comparison between the CSAG and CCUK studies. It is striking that there were significantly more five-year-old children with normal speech in the CCUK data set. It is also of note that there were approximately 20% of children in both the CSAG and CCUK studies who were in the worst categories of intelligibility. This grouping is consistent with other literature where around 20% of children have persistent speech disorders 34,35.

**Figure 2:**
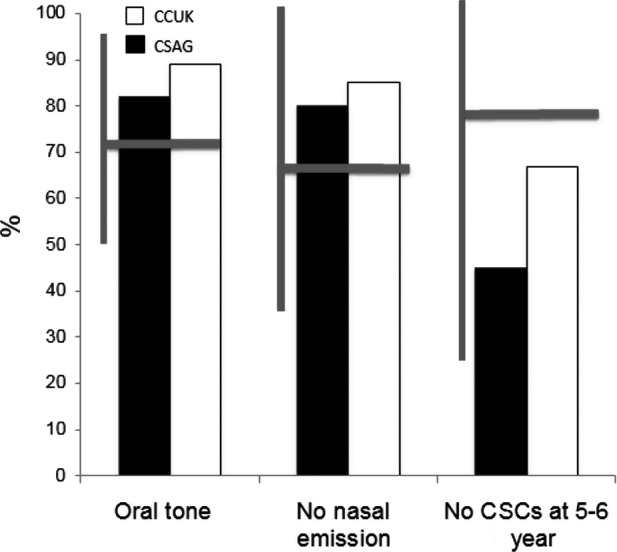
Summary of 34 studies where recordings of children with cleft palate were reviewed [median and range shown 38,39]. The horizontal bar is the median, and the vertical bar is the range of estimates across studies. The data from the CSAG (white bars) and CCUK studies (black bars) are also presented thus enabling comparison with previously reported values in the literature.

Comparison with previous studies is always difficult especially where methodological differences exist. Lohmander 36 undertook a comprehensive critical review of published studies reporting speech outcomes at 5 years of age. Studies often lacked information about ages at assessment, reporting of inter- and intrarater reliability, details of the assessor and variable speech samples. Frequently, there were small heterogeneous samples and selection or exclusion bias. Twelve of the studies reported speech outcomes at 5 years of age and one-third of these patients were in the original CSAG study. Figure2 shows that in those studies analysed using speech recordings there is a wide range of outcomes for each of the parameters, but the CCUK study is above the median for the parameter of no hypernasality (i.e. oral tone) and no nasal emission. It remains below the median for cleft speech characteristics, but this is almost certainly because narrow phonetic transcription, recognized as the gold standard, was used in both the CSAG and CCUK studies. Otherwise, there are no recent data to compare with the CCUK speech outcomes.

### Approaches to analyses

Given that fifteen years separated the two studies, it is inevitable that the two data sets were evaluated using different but linked tools. The CSAG data were evaluated using an early version and non-validated assessment – CAPS – the Cleft Audit Protocol for Speech (Harding, personal communication) and a modification of the Eurocleft Speech study for articulation 28. Once the CSAG Report was published, it was apparent that a validated speech outcome tool was needed. Over the next 5 years, the CAPS-A was developed and tested for its reliability, validity and applicability 2. This has been adopted as the UK’s national audit outcome tool and has been used in many studies since 37–39. In addition, the Americleft Speech Group has adopted this tool for their outcome reporting 29.

Although there were many similarities between the CSAG and CCUK studies, there were differences at the level of detail. The parameters and the use of narrow phonetic transcription were common to both tools. The rating scales of intelligibility/distinctiveness, voice and grimace were similar too. Although each tool included scales for rating hypernasality, hyponasality, audible nasal emission and nasal turbulence, there were differences. The CAPS scales were all 5-point scale in length. The CAPS-A scales varied between 5-point and 3-point scales, but importantly, each parameter and scalar point were defined, and listeners used these to determine their rating. With regard to articulation, the approach taken using cleft speech characteristics and summary categories was similar. However, the scoring systems for the articulation features differed considerably. In the original CSAG data, targets were scored on a 3-point scale of correct, almost correct and incorrect. No account was taken of frequency of errors. In CAPS-A, scoring is on a 3-point scale and reflects the number of consonants affected by a cleft speech characteristic and therefore provides a measure of severity.

To make the comparisons between the two data sets, the data have been compared using a presence criterion for features of hypernasality, hyponasality, nasal emission and nasal turbulence. For each CSAG 5-point scale, the scalar points 2, 3 and 4 have been summed to reflect presence of the feature. Articulation has been compared according to presence of individual cleft speech characteristics and speech categories.

### Process issues

Recognized as essential in comparative studies 17,40–45, the core speech sample was the same, except for the addition of three extra sentences to the CCUK speech sample, and therefore, this essential methodological requirement has been met.

Both the CSAG and CCUK analyses were based on audio–video recordings by two trained listeners. The nature of the recording differed across the two studies. In CSAG, these were analogue recordings but digital in CCUK. Shriberg et al. 46 compared digital and analogue audio recording systems and noted a trend for the digital samples to be scored more critically compared to the analogue samples. This suggests the improved speech results are not associated with the nature of the recording medium. Indeed, it had been thought that the advent of digital video camcorders would improve the quality of recordings obtained in the CCUK study. There was however a very wide variation in quality of recordings obtained, which is likely to be a result of the large number of different SLTs undertaking the task. Furthermore, the extent to which the differences in recording equipment used across the centres, detailed in Appendix S1, has influenced the findings is unknown. The CSAG study, by contrast, used two research SLTs to gather all the recordings of the cohort, using identical recording equipment. Headphones were worn by the SLTs which enabled them to check the quality of recording and correct recording errors immediately. Although this was stated as part of the methodology for the CCUK study, it was evident this was not always undertaken. Future studies, involving multiple data collectors, should ensure that all the SLTs are very familiar with the methodological guidelines, and some method of checking adherence needs to be devised 25.

There were other differences; a structured listening protocol was developed for use with CAPS-A in CCUK but not in CSAG. This enabled the rating of particular parameters on specific parts of the speech sample 25 and was developed to address the known difficulty of simultaneously assessing multiple parameters 47. The CAPS-A tool also has a specifically developed training programme which users complete prior to using the tool 25.

### Reliability

Reliability studies were reported for both studies. For the CCUK study, although the intrarater reliability was satisfactory, the inter-rater reliability was disappointing and in the main lower than for previous data sets 25,29. However, percentage agreement scores compare favourably with other studies. Lohmander and colleagues 27 reported the mean exact intrarater agreement for combined 5- and 7-year data to be 95% (88–97%) for one rater and 93% (84–98%) for the other. Mean inter-rater agreement for CCUK was 85% (62–95%). It is also likely that some of the CCUK scores are associated with an anomaly that has been found with the kappa formulae in some data sets 7,29,42,48. Chapman et al. 29 reported that if there is insufficient variability in the speech parameters, such that scores cluster in one corner of the cross-tabulation table for categorical ratings, or the range of ratings is very narrow for continuous ratings, the resulting kappa will tend to be smaller 44. This may explain why agreements for the parameters of passive and non-oral are poorer associated with their low prevalence. Lower agreement has been consistently found on the anterior summary category, and this may reflect previous findings associated with palatal/palatalization and lateral/lateralization 25,29. Studying the raw data in detail revealed how there appeared to be a considerable difference in the coding of palatalization. Listener 1 usually categorized this as a cleft speech characteristic, in contrast to Listener 2 who categorized this more typically as a non-cleft speech immaturity 46. This may be because the category non-cleft speech immaturities was not given sufficient emphasis in training 29. Notwithstanding, with such a large data set and the time that analysis takes, a consensus listening approach or a panel of judges was not possible 48, so the pragmatic approach of using one listener with excellent intrareliability was agreed by the research team. However, this remains one of the main limitations of this study. It is recommended that further study of those participants in which there was more than one scalar point difference between listeners on the different parameters should take place.

This study is the first report of speech outcomes from the CCUK study, and we have restricted this to perceptual speech findings. Further work will be undertaken to identify factors which may account for the variation in outcomes and the influence of centre effects, surgery (including timing and type of palate repair), velopharyngeal insufficiency and secondary speech surgery, fistulae, hearing, therapy and social patterning. The extent to which timely secondary speech surgery had already taken place is important in understanding the structurally related speech outcomes reported here. The group with very poor outcomes on the intelligibility/distinctiveness scale needs to be studied in detail. Early prediction and appropriate intervention would be key to targeting resource allocation in cleft healthcare models.

## Conclusion

Centralization of cleft care in the UK over the last fifteen years appears to have resulted in improvements in speech outcomes in five-year-olds born with unilateral cleft lip and palate. Part of this improvement may be associated with the development of multidisciplinary teams within the cleft centres. The identification of speech problems in about 20% of these children at school entry needs further study to better understand and identify where resources should be allocated. Speech outcomes reflect the outcomes of interdisciplinary team working and not the specialty alone.

## Clinical relevance

Centralization of cleft services in the UK over the last fifteen years has reduced the number of cleft centres from 57 to 11. Higher volumes of patients are now treated by an expert workforce. A key outcome in children born with a cleft palate is speech. In the previous CSAG study, this outcome was poor, but the implementation of centralized multidisciplinary care appears to have resulted in speech outcomes which were better. A percentage of children still had very poor speech, and this percentage remained the same in both dispersed and centralized care models.

## Supporting Information

**Appendix S1.** Audio–Video Recording Equipment used in the CCUK Study by Centre.
