# Supplementary material for: The Cleft Care UK study. Part 4: perceptual speech outcomes
Source: Orthod Craniofac Res. 2015 Nov 16;18(Suppl 2):36–46. doi: 10.1111/ocr.12112 (PMC4670716; doi:10.1111/ocr.12112)
Supplement: Supplementary file 1 [file ocr0018-0036-sd1.docx]

**Appendix 1** Audio–Video Recording Equipment used in the CCUK Study by Centre

| **Centre** | **Camera and microphone** |
| --- | --- |
| West Midlands (2 cameras) | 1. Sony 3CCD 150X internal microphone  2. Zoom HD; Rode external microphone |
| Northern and Yorkshire, Leeds | Sony HDR-XR16; internal microphone |
| Northern and Yorkshire, Newcastle | Panasonic HDC-HS300 Sony ECM-MS907 |
| Northwest England, Isle of Man, North Wales Liverpool | Panasonic 3CCD; Rode NT3 microphone |
| Northwest England, Isle of Man, North Wales Manchester | Panasonic NV-GS180; Rode NT3 microphone |
| South Wales South West, Swansea | JVC HD GY-HM100E with RODE NTG-2 microphone |
| South Wales South West, Bristol | Panasonic NV-GS75; Rode external microphone |
| South Thames | Sony HDV 1080i Mini DV; Rode NT3 microphone |
| Cleft NetEast | Sony HDR-HC9 HDV 1080i; RODE NT3 microphone |
| West of Scotland, Glasgow | Canon Legria FS200; internal microphone |
| Spires, Salisbury | Sony digital DCR-DVD91E; Rode NT3 microphone |
| Spires, Oxford | Canon Legria FS21; internal microphone |
| Northern Ireland | Sony DCR-DVD100E; Sony ECM MS907microphone |
| North Thames,  St. Andrew’s Centre  Great Ormond Street Hospital for Children | Panasonic NV-MX8B Sony ECM- 55B; lapel clip microphone  Sony DCR-TRV900E; Rode NT3 microphone |
| Trent | Canon XM2 3CCD; Sony ECM MS907 microphone |
| East of Scotland, Edinburgh | Sony DCR-VX2000E; Sennheiser ew100G2 microphone |
